# Lime Pretreatment of Miscanthus: Impact on BMP and Batch Dry Co-Digestion with Cattle Manure

**DOI:** 10.3390/molecules23071608

**Published:** 2018-07-02

**Authors:** Hélène Laurence Thomas, Jordan Seira, Renaud Escudié, Hélène Carrère

**Affiliations:** LBE, University of Montpellier, INRA, 102, Avenue des Etangs, 11100 Narbonne, France; helene.thomas@inra.fr (H.L.T.); jordanseira@hotmail.fr (J.S.); renaud.escudie@inra.fr (R.E.)

**Keywords:** anaerobic digestion, biogas, lignocellulosic biomass, alkali pretreatment

## Abstract

In Europe, the agricultural biogas sector is currently undergoing fast developments, and cattle manure constitutes an important feedstock. Batch dry digester processes with leachate recirculation prove to be particularly interesting for small-scale plants. However, their startup being relatively slow, the process could be facilitated by co-digestion with energy crops. In this study, *Miscanthus x* *giganteus* was chosen for its high biomass yields and low input requirements. The carbohydrate accessibility of this lignocellulosic biomass is limited but may be improved with alkali pretreatment. The efficiency of lime (CaO) pretreatment with low water addition on the biochemical methane potential (BMP) of miscanthus was investigated through two experimental designs (CaO concentrations ranged between 2.5 and 17.5% and pretreatment lasted 1, 3, or 5 days). The pretreated miscanthus was then co-digested with cattle manure in dry leach bed reactors. CaO pretreatments led to a 14–37% improvement of miscanthus BMP, and a 67–227% increase in the first-order kinetics constant; a high contact time was shown to favor methane production. According to these results and to industrial requirements, miscanthus was pretreated with 5 and 10% CaO for 5 days, then co-digested with manure in dry leach bed reactors. Nevertheless, the promising results of the BMP tests were not validated. This could be related to the high water absorption capacity of miscanthus.

## 1. Introduction

Within the context of having to mitigate global warming and reduce greenhouse effect gas emissions, anaerobic digestion (AD), which allows the production of renewable energy from various organic wastes, is undergoing rapid developments. In particular, the French government has set the target of 1500 biogas plants by 2020, including 1000 plants based on agricultural feedstocks [1]. In many agricultural anaerobic digestion plants, manure represents the main part of the feed. Furthermore, cattle manure is available in high quantities all over the country. Its production has been estimated at about 69 MT per year in 2010 [2]. Cattle manure is rich in straw and is characterized by a total solids (TS) content of about 20–30%. It is thus suited for dry AD [3], also called solid-state anaerobic digestion. A process occupying an important part in the development of the agricultural AD sector is the leach bed reactor (LBR) operated in batch mode [4]. In this high-solids process, the solid substrate is loaded into the reactor while a liquid phase, usually stored in a separate container, is regularly sprinkled over the solid bulk and percolates through it. However, batch mono-digestion of cattle manure usually takes time to start up and produces low amounts of biogas [5].

In this view, co-digestion would be a good option for improving biogas production and productivity. For example, Botji et al. (2017) [6] demonstrated how the co-digestion of poultry manure with maize silage improved methane production by 24% relative to mono-digestion. This is presumably due to the improved C/N ratio. Nevertheless, the use of food or feed product-dedicated crops (e.g., cereals) or energy crops as AD plant feedstocks is limited to less than 15% of the total feed ration by the French national legislation [7]. Some exceptions are, however, still possible for catch crops and biomass cultivated on marginal lands that are not in conflict with food and feed production. Among these, miscanthus presents many advantages, including high biomass yields, low input requirements (i.e., water, fertilizers), prolonged soil cover, reduced soil disturbance, and increased soil carbon content [8,9]. This crop can also grow on polluted soils [10,11]. Few studies have used *Miscanthus x giganteus* as a co-substrate for manure AD. Moiceanu et al. (2016) [12] used miscanthus as a reference co-substrate to investigate the influence of different types of manure on biogas production.

Nonetheless, for most lignocellulosic biomasses, carbohydrate accessibility is limited and AD performance can be improved by pretreatment [13]. For example, a 170 °C hydrothermal pretreatment of miscanthus led to a 21% increase in biogas production [14]. In another study, Nges et al. (2016) [15] applied grinding, steam explosion, and acid and alkali pretreatments to *Miscanthus lutarioriparius*. The best result—i.e., 57% increase in methane production—was obtained with a mild alkaline pretreatment. Indeed, a high lignin content and lignin/polysaccharides links have been identified as main bottlenecks for lignocellulosic biomass AD [16]. Among the different kinds of pretreatment techniques (mechanical, biological, chemical) [17], alkali pretreatments have been recognized as the most efficient for degrading lignin [18,19]. Alkali pretreatments generally employ soda. Because digestates from agricultural AD plants are systematically used as organic fertilizers and returned to agricultural soils, sodium spreading into soils should be avoided. The aim of this study is to therefore investigate miscanthus alkali pretreatment with lime.

The first objective was to assess and optimize miscanthus pretreatment conditions compatible with a further application in dry AD (i.e., with low water content). In fact, high solid content pretreatments reduce waste generation, do not require a separation step before further processing, and reduce the environmental impact of the entire process [20]. In order to keep pretreatment costs as low as possible, the conditions were set to ambient temperature. The impact of lime concentration and pretreatment duration on the biochemical methane potential (BMP) of miscanthus was investigated using a response surface methodology. The second objective of this study is to evaluate the impact of a selected lime pretreatment of miscanthus on its batch co-digestion using cattle manure in an LBR. Startup performances, as well as methane production, are reported.

## 2. Results

### 2.1. Impact of Lime Pretreatment on BMP

The different pretreatment conditions were carried out at a TS content of 13%, CaO concentrations between 2.5 and 17.5%, and pretreatment durations of 1, 3, or 5 days. Two experimental designs were created consecutively: Design 1 (CaO concentrations between 7.5 and 17.5%, and durations of 1, 3, or 5 days) and Design 2 (CaO concentrations between 2.5 and 12.5%, durations of 1, 3, or 5 days). BMP tests were performed in duplicate using these pretreated substrates. Table 1 reports the BMP and first-order kinetics constant k values. The duplicates revealed a very good repeatability. In comparison with the BMP obtained for the non-pretreated biomass (158 ± 2 NmL_CH4_·g_VS_^−1^), the effect of the pretreatment was significant (*p*-value = 9.8 × 10^−4^) and positive. An improvement in BMP was observed, ranging from +14% (for 15% CaO; 1 day) to +37% (for 5% CaO; 5 days) for the best-performing condition.

The adjustment of Equation (2) for estimating k was excellent over all experimental conditions (R² > 0.97; data not shown). With a focus on the first kinetics constant k, a strong and positive effect was also noticed as an improvement, ranging from +63% (for 10% CaO; 1 day) to +221% (for 17.5% CaO; 3 days). This was calculated by comparing k with that obtained for raw biomass (0.024 ± 0.002 NmL_CH4_·g_VS_^−1^·d^−1^). Even though a clear correlation did not emerge, the evolution of BMP and k presented similar trends, as the highest BMPs were mostly characterized by the highest k values. These results suggest that lime pretreatment of the miscanthus does, indeed, induce an increase for both BMP and k. Despite these positive observations, it was not possible to assess which parameter was most relevant. The effect of each parameter therefore needs to be unraveled using an experimental design. 

### 2.2. Mathematical Models to Describe Impact of Concentration and Pretreatment Duration on BMP Values

#### 2.2.1. Experimental Design 1 (CaO Concentration from 7.5 to 17.5% and Duration from 1 to 5 Days)

The effect of variables A (CaO concentration) and B (pretreatment duration) on BMP and first-order kinetics constant k was investigated by statistical analysis based on response surfaces. According to Oliveira et al. (2015) [21], response surface methodology (RSM) is a collection of both mathematical and statistical techniques that involves (i) designing and carrying out experiments with a reduced investment; (ii) building models; (iii) evaluating the relative significance of the studied variables; and (iv) assessing the optimal conditions for a favorable response. Using data displayed in Table 1 and multiple regression analysis, a polynomial equation was determined to predict BMP and k depending on the variables, as well as their interactions (Equation (1)). The different coefficients with their standard deviation, the Fisher value (F-value), and the coefficient of determination R² of the models are provided in Table 2 for each design. 

The F-value for each case was far less than the Fisher parameter calculated at the 95% confidence level (161.45), thus indicating that the models were significant and fitted the data nicely. Joglekar and May [22] suggested that, for a good fit of a model, the R² should be higher than 0.8. The high R² (≥0.95) obtained for each case was a strong hint of suitability, as it indicated that 95% of the data were explained by the regressions, even reaching 99.9% for the prediction of k in Design 1. Consequently, all the models were validated. 

**Figure 1:**
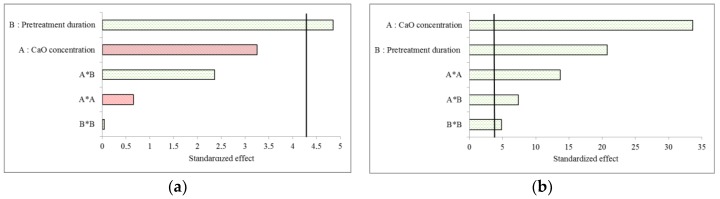
Pareto diagram showing the effect of different coefficient terms on BMP (**a**) and kinetics constant k (**b**) for Design 1. Red bars indicate a negative impact, and green bars show a positive impact. Bars exceeding the vertical line point to the significance of the coefficient terms (*p* < 0.05, corresponding to 4.3 according to Student t-test in our conditions).

Standardized Pareto charts displaying the effects of the different terms of the models are provided for BMP in Figure 1a and for the first-order kinetics constant k in Figure 1b. The duration of the pretreatment (variable B) was the only parameter that significantly affected the BMP (Figure 1a). Moreover, the effect being positive, long contact times between CaO and miscanthus should favor methane production. Interestingly, the pretreatment duration also produced a very significant and positive effect on the first-order kinetics constant k, although this effect was less significant than the effect of CaO concentration (Figure 1b). In addition, both quadratic terms proved to be significant for the first-order kinetics constant k, thus implying that the influence of the variables was not necessarily linear. Finally, the interaction term A × B was also significant, with a positive effect on the first-order kinetics constant. This result could not have been anticipated by using a univariate approach. Although both BMP and k were related, it is noteworthy that both selected variables affected these responses at different levels (positive or negative effect) and with various extents of contribution.

**Figure 2:**
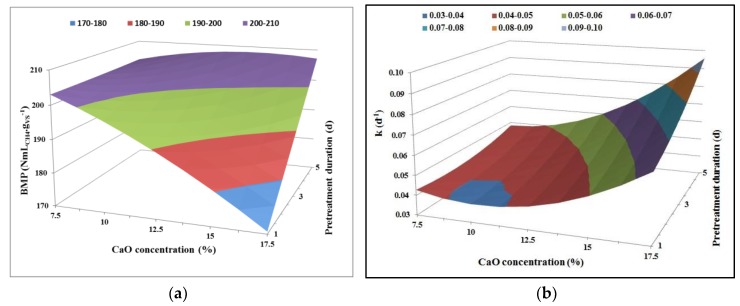
Response surface plots showing the impact of lime concentration and pretreatment duration on BMP (**a**) and kinetics values (**b**) for Design 1.

Response surfaces were plotted in 3-D for each parameter (i.e., BMP and k) as a function of CaO concentration and pretreatment duration (Figure 2). The response surface plot for BMP (Figure 2a) led to the following observations: (i) the longer the duration of the pretreatment, the better the BMP, which is the same conclusion as that stated in a previous section; (ii) the more the CaO concentration increases within the experimental domain, the more the BMP decreases. Even if it was not significant, the negative effect of this variable could be anticipated from Figure 1. Consequently, in order to favor methane production, a combination of “low” CaO concentration (lower part of the experimental domain) with “high” contact time for pretreatment (upper part of the experimental domain) could be a viable option. 

Focusing on the response surface plot for k (Figure 2b), the coupling of a “high” CaO concentration with a “long” pretreatment duration was linked to a high methane production rate. Nevertheless, the effect was more pronounced in the upper part of the domain (CaO concentration > 16% and pretreatment duration > 3 days) where few experimental data were generated (Table 1). The prediction thus rather relies on extrapolation rather than on interpolation. Therefore, the selection of the experimental domain to be exploited was made where the trend described by the model is well established. In this case, it is the middle-upper part of the domain that was chosen. Application of a “medium” CaO concentration with a “long” contact time pretreatment was hence considered most relevant to achieve a high first-order kinetics constant k. 

As the responses for BMP and k reflect different impacts, it is difficult to find a consensus regarding the values of the variables to select (lime concentration and pretreatment duration) in order to optimize both parameters simultaneously. Moreover, no optima could be determined within the investigated domain. Only one extreme stationary point was identified in the lower domain for the kinetics constant k, which is irrelevant when both BMP and k need to be maximized. Owing to its energetic relevance, BMP is the parameter to prioritize in this study. The first-order kinetics constant k will therefore not be discussed anymore in the further section. 

#### 2.2.2. Experimental Design 2 (CaO Concentration from 2.5 to 12.5% and Duration from 1 to 5 Days)

According to the trend displayed in Figure 2a, the application of a lower CaO concentration could contribute to enhancement of the BMP and even lead to an optimal result. The experimental domain was thus extended to CaO concentrations ranging from 2.5 to 12.5% for a second design of experiments (DOE). Finally, and even though the duration of pretreatment had a positive effect on BMP, its experimental domain was not extended for the following reasons: (i) longer contact times would not be realistic for applications in full-scale plants; (ii) the extension of a Doehlert design with the reuse of certain points is only possible for one factor; (iii) the possible formation of refractory compounds, which could further impede methane production [23]; and (iv) the possible pre-degradation of accessible substrates by microorganisms already present in the bulk matrix, which could decrease the bioavailable fraction for methane production [24].

**Figure 3:**
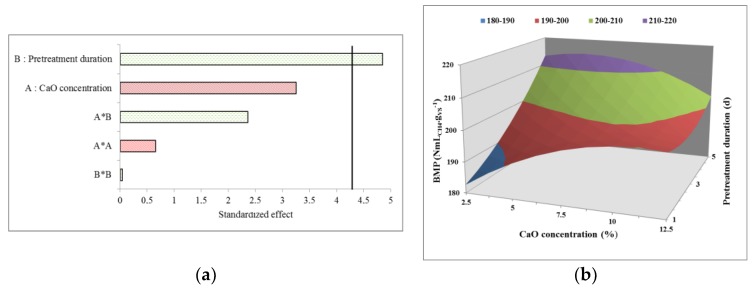
Pareto diagram showing the effect of different coefficient terms on BMP (**a**) and response surface plot showing the impact of lime concentration and pretreatment duration on BMP in Design 2 (**b**).

The results obtained for the second DOE are summarized in Table 2, and as a Pareto chart and response surface plot in Figure 3a,b. As depicted in Figure 3a, variable B (pretreatment duration) was the only one that significantly and positively impacted methane production, as was previously observed. The response surface (Figure 3b) confirmed this observation, as BMP increased with the application of longer contact times. Regarding variable A (CaO concentration), the effect was minor within the investigated domain (from 2.5 to 12.5%), even though the highest concentrations appear to lead to an increase in BMP. Unfortunately, it was not possible to determine an optimum in the second DOE domain. However, high responses for BMP can be observed in a region of interest. According to these results, the application of a long contact time seems necessary for enhancing methane production. A duration of 5 days for the “pretreatment duration” variable was thus selected. For the CaO concentrations, response surfaces revealed that the additional increase in BMP was negligible when a concentration above 10% was applied, while a detrimental effect was even possible for concentrations below 5% (Figure 3). 

### 2.3. Co-Digestion

According to RSM conclusions and due to economic incentives, a concentration of 5% CaO was first selected. In order to confirm the trends displayed by RSM (e.g., increase in k with increasing CaO concentration), it was also worthwhile to consider a higher CaO concentration (10%). As a consequence, the following combinations were retained for application in an LBR at lab scale: 5% CaO during 5 days of pretreatment and 10% CaO during 5 days of pretreatment (performed in duplicate). 

**Figure 4:**
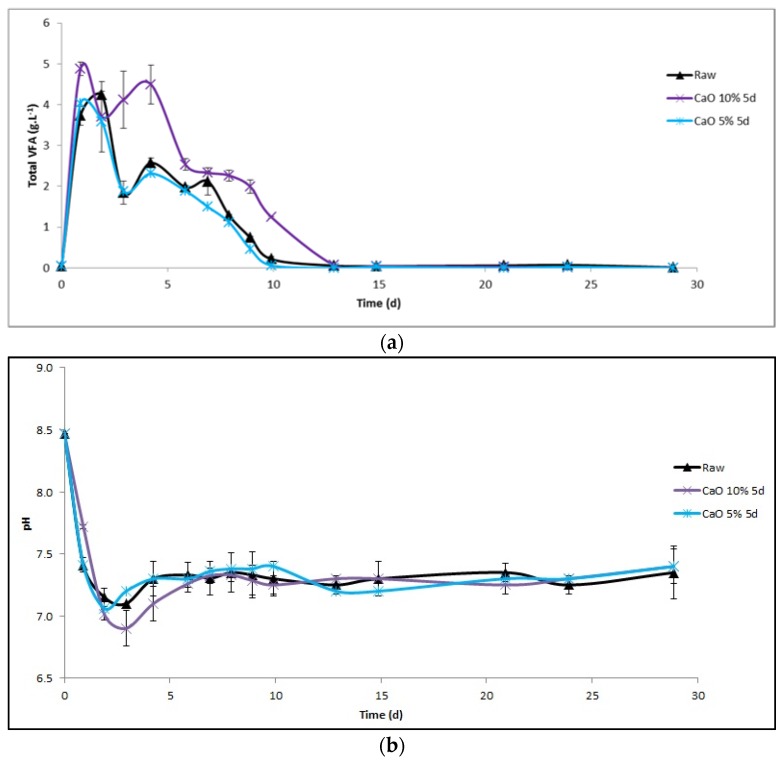
Volatile fatty acid (VFA) concentration (**a**) and pH variation (**b**) in leachate during the first 30 days.

The concentration in volatile fatty acids (VFAs) in the leachate and the evolution of pH were measured each day at the beginning and on a regular basis thereafter (Figure 4). For duplicates, the VFA concentrations were similar (Figure 4a). VFAs were rapidly produced, and their maximum value was reached after 1 day for miscanthus pretreated with 5 and 10% of lime for 5 days (4.5 ± 0.5 g·L^−1^ and 4 g·L^−1^, respectively). For raw miscanthus, the maximum was reached after 2 days (4.2 ± 0.1 g·L^−1^). For all reactors, pH variations were similar (Figure 4b). The evolution of VFA and pH can be separated into two steps. During the first step, the accumulation of VFA during the first 3 days induces, with a brief delay, a slight decrease in pH down to 6.8. Two hypotheses could explain this observation: either (1) the positive impact of higher alkalinity due to CaO or (2) the buffer capacity of manure. The second hypothesis, manure buffer capacity, seems to be more plausible, given that in the case of co-digestion with the raw substrate, the pH is similar to that of the pretreated substrate. The pH did not present a sharp drop and the VFA concentration was not very high either. Moreover, since the VFA/alkalinity ratio remains below 1 (Table 3), the risk of acidification is avoided [25] and the drop in pH is swiftly reduced. During the second step, the pH stabilized close to 7.3 due to the decrease in VFA concentrations. After 15 days, there were no more VFAs accumulated; they were simultaneously produced and consumed at the same rate. 

Methane production from co-digestion of raw miscanthus and miscanthus pretreated with lime at 5 and 10% for 5 days with cattle manure was 158 ± 4, 150, and 167 ± 2 mL·g_VS_^−1^ after 59 days, respectively (Table 3). The 59-day period was selected because it corresponds to the duration of a batch in industrial plants. For this time span, there is no significant difference in the methane production between the control and the two different conditions of pretreatment (*p*-value = 0.7, 0.82) nor between both pretreatments (*p*-value = 0.82). However, after a shorter time, differences can be observed (Table 3). After 15 days of anaerobic co-digestion in an LBR, the methane production was higher for the miscanthus pretreated at 10% than for the raw miscanthus (+18%). It is related to a higher kinetics constant (+23%).

## 3. Discussion

### 3.1. BMP and Pretreatment

The BMP value of unpretreated miscanthus was 153 ± 7 NmL_CH4_·g_VS_^−1^. This value lies within the lowest range of published miscanthus BMP values (170 mL_CH4_·g_VS_^−1^ [14] to 227 mL_CH4_·g_VS_^−1^ [26]). This low methane potential is most certainly linked to the high lignin content of the Floridulus clone [27]. Alkaline pretreatment may therefore be relevant for improving methane production from this clone.

Lime pretreatments have been far less studied than NaOH pretreatments, although sodium has detrimental effects on agricultural soils when digestates are used as biofertilizers. In addition, miscanthus has been scarcely employed in AD studies, which are extensively dedicated to agricultural residues such as rice straw, sugarcane bagasse, corn stover, wheat straw, or other energy crops, such as switchgrass [28]. To the best knowledge of the authors, there has not yet been any study on the impact of lime pretreatment on *Miscanthus x giganteus* BMP. Moreover, what makes this study innovative is that low inputs were set for the pretreatment conditions (low water input with high solid concentration and no heat energy input with ambient temperature conditions). In particular, for humidifying the entire biomass, a minimum amount of water was used. This corresponds to only 13% TS due to the high absorption capacity of miscanthus. Peces et al. (2015) [29] clearly demonstrated that total solids content is a significant parameter for the performance of sonication pretreatment, although it has been rarely considered in pretreatment optimization procedures. However, the doses of lime are consistent with those applied to other types of herbaceous biomass. For example, Jiang et al. (2017) [28] pretreated giant reed biomass with 1, 3, 5, 7, 12, and 20% (g_Ca(OH)2_·g_initialTS_^−1^) at 25 °C for 24 h. They observed an increase in methane yields between 7 and 34%. Another study obtained a 23% improvement of BMP with sunflower straw pretreated at 30 °C with 4% (g_Ca(OH)2_·100g_TS_^−1^) for 1 day [18].

Although good performances have been achieved with lime, compared to potassium and sodium hydroxide at the same molarity, it has proven to be significantly less efficient for delignification [30]. Results indicate that with an equivalent molar basis of OH^−^, potassium and sodium hydroxide have a performance that is superior to calcium hydroxide [30]. 

The efficiency of pretreatments also depends upon the substrate. Indeed, a low lignin content is the main factor in promoting enhanced enzymatic saccharification [19] or enhancing anaerobic digestion [16]. Miscanthus is also widely used as animal bedding due to its high absorption capacity [31]. Thus, for an equivalent biomass TS content, less free water would be available with miscanthus than with other types of biomass. These, associated with a low lime solubility (1.65 g·L^−1^ at 20 °C, corresponding to 5.5% in this study), could reduce the amount of lime in contact with the substrate. In addition, Boix et al. (2016) [32] demonstrated that the absorption capacity of miscanthus increases with alkaline treatment. This can be explained by the removal of hydrophobic compounds, due to more exposed OH groups from cellulose or hemicellulose on the stem surface.

### 3.2. Pretreatment and Co-Digestion

Pretreatments are a promising solution in BMP test series. However, the performances in the LBR could not be confirmed if the methane production was estimated at 59 days. The BMP of manure used for the experiment was 202 ± 30 NmL_CH4_·g_VS_^−1^, which was higher than the BMP of raw miscanthus and within a similar range to pretreated miscanthus BMP. The maximum expected methane production with a ratio of 40%_VS_ miscanthus and 60%_VS_ manure is presented in Table 3. While 87% of the expected methane production was reached after 59 days co-digestion with raw miscanthus, 75 and 80% of expected methane production were obtained with miscanthus pretreated at 5 and 10%, respectively. Riggio et al. (2016) [4] carried out the process using an LBR fed with spent cow bedding. They obtained 168 NmL_CH4_·g_VS_^−1^ after 60 days, which represents 86% of the BMP value. Thus, the overall performance of the LBR evaluated in this study is satisfactory, although the small increase in methane production at 59 days remains surprising. Dry anaerobic digestion inoculum might require some adaptation to the pretreated biomass. Another explanation could be the high water absorption capacity of miscanthus. As BMP tests were carried out in diluted medium (5 g_TSmiscanthus_·L^−1^), the high amount of available water can favor contact between lime and biomass. This could enhance the action of lime if it continues during the AD process.

The improvement in methane production after a 10% pretreatment was quite low (6%). This was related to a higher VFA production at the beginning of the AD run (Figure 4a), thus revealing that, unlike a 5% pretreatment, the 10% pretreatment can lead to the release of easily biodegradable matter. Nevertheless, this increase in methane production is not sufficient to justify the application of a full-scale pretreatment.

## 4. Materials and Methods

### 4.1. Miscanthus

*Miscanthus. x giganteus* Floridulus was grown in the North of France (49°53 N, 3°00 E) [27] at the INRA experimental unit of Estrées-Mons and harvested in winter 2015 during its eighth year. The soil is a deep loam soil (Orthic Luvisol, Roma, Italia, FAO classification). The climate is oceanic. The stems were dried at 64 °C for 4 days in a ventilated oven and ground with a crusher (Viking, model GE 220, STIHL, Stuttgart, Germany) to a coarse size (around 6 cm). The TS and volatile solids (VS) content were 94% and 98%_TS_, respectively.

### 4.2. Experimental Design 

To assess the effect of CaO pretreatment on BMP and the first-order kinetics constant k, a two-factor Doehlert-type uniform network was used to define the experimental matrix. The principle and strengths of such a design is described by Goupy et al. (2014) [33] and by Witek-Krowiak et al. (2014) [34]. Briefly, it consists of a two-variable (z = 2) Doehlert design and requires N = z² + z + C experiments, with z as the number of variables and C as the number of center points. Here, N was equal to 2² + 2 + 1 = 7. The center point was repeated once. As the experiments were performed in duplicate for each condition, the total number was 16. The two variables of interest (called factors) were defined as the CaO concentration applied for pretreatment (variable A) and the duration of pretreatment (variable B). The ranges to be studied for both factors were selected based on literature, sound reasoning, and preliminary experiments carried out at the laboratory. Thus, for variable (A), the range was between 7.5 and 17.5% (% mean g_CaO_ per 100 g_TS_). For variable (B), the range was between 1 and 5 days. As no optimum was found within the investigated domain, the range for variable (A) was extended to a second set of experiments and defined between 2.5 and 12.5%. The experimental domains, expressed as coded (±0, 0.5, 0.866, and 1) and real values for each factor, are listed in Table 1 for both designs. 

A full second-order polynomial equation was used to model the values obtained for BMP and the first kinetics constant k as a function of the applied lime concentration (A) and the duration of the pretreatment (B). The system can be described by the following equation (Equation (1)):

(1) $$Y=a_{0}+a_{1}A+a_{2}B+a_{12}\mathrm{AB}+a_{11}A^{2}+a_{22}B^{2}$$

where Y is the BMP or the first-order kinetics constant k, a_0_ is the constant term, a_1_ and a_2_ are the linear coefficients associated with each variable, a_12_ is the coefficient associated with the interaction between both variables, and a_11_ and a_22_ are the quadratic coefficients. A detailed calculation of the coefficients is already available in the literature [35].

The model was validated using a Fischer test. The significance of each coefficient in the model was tested using a Student’s *t*-test [35]. The results were then compared using standardized effects in a Pareto chart. 

### 4.3. Alkaline Pretreatments

The pretreatments were carried out at ambient temperature, without mixing, and in duplicate in 500 mL flasks using lime (CaO, Akdolit^®^ Q90; purity ≥ 92%, Paris, France) and 2 g_TS_ of miscanthus in conditions reported in Table 1. Another originality of this study is the high TS loading (130 g·L^−1^) selected to test conditions with low water input. 

### 4.4. Measure of Methane Potential

All pretreated samples (solid and liquid fractions altogether) were digested in a 500 mL flask with a working volume of 400 mL. Bicarbonate buffer (NaHCO_3_, 50 g·L^−1^), macroelement and oligoelement solutions, anaerobic sludge at 5 g_VS_ L^−1^, and the substrate at 5 g_TS_·L^−1^ were added [36]. Degasification with nitrogen was carried out to obtain anaerobic conditions. Duplicate bottles were incubated at 35 °C for 60 days. 

### 4.5. Methane Production Kinetics

All methane potentials are expressed in NmL_CH4_·g_initialVS_^−1^. Thus, the eventual losses of organic matter during pretreatments are included in the results. To quantify the impact of pretreatment on the kinetics of methane production, the first-order kinetic constants were calculated by using the least-squares fit of methane production data versus time (*t*) to the following equation:

(2) $$V=Vmax\left(1-e^{-kt}\right)$$

where *V* is the volume of methane (NmL_CH4_·g_VS_^−1^), *V_max_* the maximum producible methane volume (NmL_CH4_·g_VS_^−1^), *k* the first-order kinetics constant (d^−1^), and *t* is the digestion time (d). *V_max_* and *k* were determined using the Microsoft Excel Solver function. 

### 4.6. Leach Bed Reactors

In order to represent farm batch dry anaerobic digestors used on farms, LBR systems were employed for these experiments. They were previously used and described by Riggio et al. [4]. Experiments were carried out in a 6 L LBR fed with 300 g_TS_ of substrate and inoculum and 1.1 L leachate. The substrate was composed of 85% (in wet weight basis) manure and 15% miscanthus (corresponding to 40%_VS_). Cow manure from wheat straw bedding was collected from a dairy farm in the South of France and stored at −20 °C. Before feeding the reactors, two different lime pretreatments were applied to miscanthus at room temperature, with low water addition (to reach 13%_TS_) and no mixing: 5 and 10% for 5 days. The inoculum used came from a previous experiment. It was composed of a mix of digested manure, miscanthus, and raw sorghum and kept at 35 °C. The leachate originated from a previous experiment and was also maintained in mesophilic conditions. It did not contain VFAs and was diluted with water and buffered with NaCO_3_ at 1.3 g·L_addedwater_^−1^. The substrate/inoculum ratio was 6 (g_VS_·g_VS_^−1^) and the TS content of the solid fraction in the reactor (including miscanthus, manure, and inoculum) was 19%. Taking the leachate volume into account, the overall TS content was 12%. The pretreated substrate at 10% CaO for 5 days and the raw substrate (control) were digested in the LBR in duplicate, whereas only one reactor was run for the other pretreatment. Degasification with nitrogen was carried out to obtain anaerobic conditions.

### 4.7. Analysis

The TS and VS contents were measured according to standard methods [37]. The leachate used was characterized in terms of alkalinity, VFA concentration, and pH. According to the APHA method, alkalinity was performed by 0.1 N hydrochloric acid titration [37]. VFAs were analyzed in a Perkin Elmer Clarus 580 gas chromatographer with helium as the gas vector [38]. The pH was measured with WTW pH-electrode SenTix 41 connected to a WTW inoLab pH 7110 operational manual transmitter. Biogas flow rate from reactors was recorded every 5 min with the use of a lab-made software connected to a Ritter Milligascounter MGC-1 V3. Biogas volume in BMP tests was monitored using a manometric device (LEO 2, KELLER) and biogas composition was determined by gas chromatography as described in Sambusiti et al. (2012) [39]. 

### 4.8. Statistical Analysis

For results obtained from the DOE, Wilcoxon tests were performed using R software (version 3.2, R Development Core Team, R: A Language and Environment for Statistical Computing, R Foundation for Statistical Computing, Austria, Vienna, 2004, ISBN 3-900051-07-0.). Effects were considered to be significant for *p*-values < 0.05.

## Figures and Tables

**Table 1:** Pretreatment conditions, biochemical methane potential (BMP), and first-order kinetics constant values and their improvement, compared to raw, for Design 1 and Design 2.

**Design 1**	**Variable A: [CaO]**		**Variable B: Duration**		**BMP**		**k**	
**Run**	**Coded**	**%**	**Coded**	**d**	**NmL_CH4_** **·** **g_VS_^−1^**	Improvement *	**d^−1^**	Improvement *
Raw	-	-	-	-	158 ± 2	-	0.024 ± 0.002	-
1	0	12.5	0	3	196	+24%	0.047 ± 0.001	+96%
1′	0	12.5	0	3	201	+27%	0.048 ± 0.001	+100%
2	1	17.5	0	3	191 ± 2	+21%	0.077 ± 0.005	+221%
3	0.5	15	0.866	5	207 ± 1	+31%	0.075 ± 0.004	+213%
4	−0.5	10	0.866	5	208 ± 4	+32%	0.051 ± 0.004	+113%
5	−1	7.5	0	3	202 ± 4	+28%	0.042 ± 0.001	+75%
6	−0.5	10	−0.866	1	199 ± 2	+26%	0.039 ± 0.002	+63%
7	0.5	15	−0.866	1	179 ± 13	+14%	0.050 ± 0.002	+108%
**Design 2**								
Raw	-	-	-	-	158 ± 2	-	0.024 ± 0.002	-
1	0	7.5	0	3	204	+29%	0.041 ± 0.001	+71%
1′	0	7.5	0	3	199	+26%	0.043 ± 0.001	+79%
2	1	12.5	0	3	199 ± 4	+26%	0.047 ± 0.001	+96%
3	0.5	10	0.866	5	208 ± 4	+32%	0.051 ± 0.004	+113%
4	−0.5	5	0.866	5	216 ± 1	+37%	0.050 ± 0.001	+108%
5	−1	2.5	0	3	193 ± 3	+22%	0.040 ± 0.003	+67%
6	−0.5	5	−0.866	1	193 ± 1	+22%	0.048 ± 0.003	+100%
7	0.5	10	−0.866	1	199 ± 2	+26%	0.042 ± 0.004	+75%

* by comparison with raw (i.e., non-pretreated) sample.

**Table 2:** Coefficients, Fisher value, and R^2^ of the two designs.

Coefficient	Design 1		Design 2
	Y_BMP_	Y_k_	Y_BMP_
a_0_	198.5 (±2.7)	0.0474 (±0.0006)	201.5 (±2.3)
a_1_	−7.2 (±2.2)	0.0173 (±0.0005)	1.5 (±1.9)
a_2_	10.7 (±2.2)	0.0107 (±0.0005)	9.5 (±1.9)
a_12_	10.4 (±4.4)	0.0076 (±0.0010)	−8.1 (±3.8)
a_11_	−2.5 (±3.8)	0.0122 (±0.0009)	−5.8 (±3.3)
a_22_	0.2 (±3.8)	0.0043 (±0.0009)	4.9 (±3.3)
F-value	1.33	0.22	0.75
R²	0.952	0.999	0.950

**Table 3:** VFA/alkalinity ratio at 3 days, methane production at 6, 10, 15, 24, 29, and 59 days, expected methane production calculated from BMP values, and first-order kinetics constants.

	VFA/Alkalinity (gHAceq·g_CaCO3_^−1^) ^1^	Methane Production (NmL_CH4_·g_VS_^−1^)							k (d^−1^)
day	3	6	10	15	24	38	59	Expected ^2^	-
raw	0.35 ± 0.07	36 ± 1	59 ± 4	78 ± 7	109 ± 3	135 ± 4	158 ± 4	181	0.040 ± 0.004
10 % 5 days	0.8 ± 0.14	43 ± 2	69 ± 3	92 ± 3	119 ± 2	145 ± 2	167 ± 2	208	0.049 ± 0.002
5 % 5 days	0.4	43	67	84	109	131	150	207	0.054

^1^ HAceq means acetic acid equivalent, ^2^ from BMP values.
